# The Ineffectiveness of Osimertinib in Epidermal Growth Factor Receptor (EGFR)-Mutated Stage IV Lung Adenocarcinoma With Bone Metastasis: A Case Report

**DOI:** 10.7759/cureus.66240

**Published:** 2024-08-05

**Authors:** Caitlyn H Livingston, Benjamin Harper, Mathew George

**Affiliations:** 1 Neuroscience, Texas A&M University, College Station, USA; 2 Medical Oncology, Calvary Mater, Newcastle, AUS; 3 Medical Oncology, Calvary Mater Newcastle, Waratah, AUS

**Keywords:** targeted treatment, adenocarcinoma lung, egfr exon 19 mutation, bone metastasis, osimertinib

## Abstract

This case report examines the effectiveness of osimertinib in a 64-year-old non-smoking female diagnosed with stage IV lung adenocarcinoma and an epidermal growth factor receptor (EGFR) exon 19 mutation, focusing on the treatment’s impact on bone metastasis. Despite initial responsiveness to osimertinib, the patient's bone lesions remained largely unresponsive, prompting a comprehensive exploration of alternative treatments and clinical trials. This report highlights the patient's clinical journey, from diagnosis through various treatment phases, culminating in palliative care, and underscores the need for further research into targeted treatments for bone metastasis in lung cancer.

## Introduction

Lung cancer remains a leading cause of cancer-related death globally, necessitating personalized and advanced treatment strategies [1]. Among the treatments available in a precision medicine landscape, osimertinib has emerged as a promising drug for managing epidermal growth factor receptor (EGFR)-mutated lung cancer [2]. Despite the drug's success, its effectiveness against bone metastasis remains underexplored and somewhat controversial. This report discusses the case of a patient with stage IV lung adenocarcinoma with an EGFR exon 19 mutation, detailing the course of the disease and the impact of various treatments, including the limited effectiveness of osimertinib on bone metastases [3].

Recent studies have shown that while osimertinib is effective for primary tumor and non-bone metastasis in many patients, its efficacy is less pronounced in bone metastasis. This results in patient morbidity with increased skeletal-related events decreasing the quality of life, negatively affecting overall survival, and posing a significant treatment challenge [4,5]. This report aims to add to the growing body of evidence that suggests that alternative strategies may be necessary for treating bone metastasis in patients with EGFR-mutated lung adenocarcinoma.

## Case presentation

The patient was a 64-year-old woman, a never-smoker, who had a complex medical history that included chronic bronchial asthma, hiatus hernia, osteoarthritis, and allergic rhinitis. Despite these challenging conditions, her health issues had been manageable until she began experiencing persistent right-sided chest pain accompanied by a worrying decline in her weight over two months. This combination of symptoms prompted her to seek medical attention. Her family history was punctuated by various cancers, though notably, there was no history of lung cancer in her family.

In June 2021, following her symptomatic presentation, a CT chest, abdomen, and pelvis was performed, revealing a mass in the left upper lobe, extensive pulmonary and thoracic nodal metastasis, and a lytic right rib lesion. A subsequent fine needle aspiration biopsy confirmed the presence of primary lung adenocarcinoma. Further diagnostic tests were undertaken, including an FDG-PET scan that highlighted increased metabolic activity in the large mass of the left upper lobe of her lungs and a lytic lesion on her right rib, suggestive of the cancer's aggressive spread.

Immunohistochemical testing provided more specificity to the diagnosis; it was positive for thyroid transcription factor-1 (TTF-1), a marker often found in lung adenocarcinoma, but negative for p40, PAX8, CK5/6, and napsin A, helping to refine the treatment focus. Also, genetic testing revealed an EGFR exon 19 mutation, a critical finding given its implications for targeted therapy, with no other common oncogenic mutations present. The overall picture was one of extensive metastatic disease, with multiple osteolytic bony metastases, complicating her symptomatic burden of disease, prognosis, and therapeutic strategy.

With this detailed understanding of her cancer's characteristics, the treatment commenced in August 2021 with osimertinib, a third-generation EGFR tyrosine kinase inhibitor (TKI), which was chosen based on its efficacy in targeting EGFR-mutated non-small cell lung cancer [6]. The initial thoracic response to this targeted therapy was encouraging, showing a significant reduction in tumor size. However, the bone lesions displayed persistent resistance to the treatment, raising concerns about the overall effectiveness of the current strategy.

**Figure 1:**
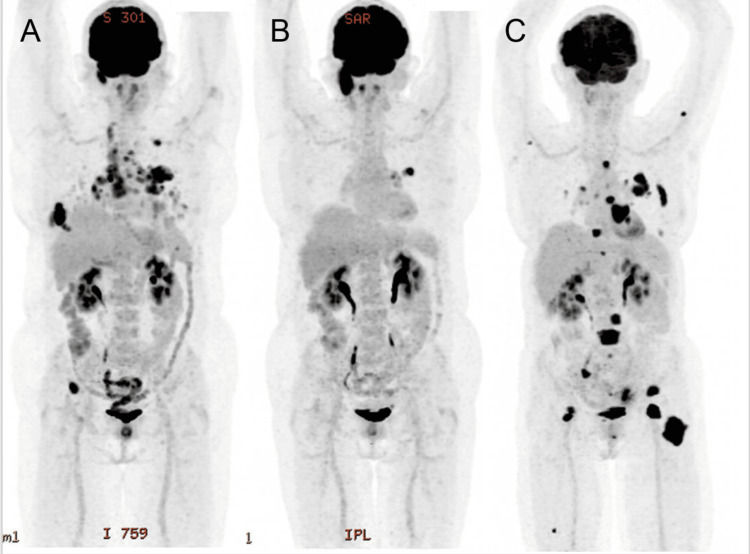
Imaging results depicting the patient's treatment course (A) The progressive response to treatment following presentation (July 2021); (B) osimertinib therapy (middle April 2022); and (C) following Taxol, carboplatin, and atezolizumab therapy (June 2023)

As the disease demonstrated progression by August 2022, despite the initial targeted therapy, the decision was made to escalate her treatment regimen after a re-biopsy of the progressive lesions, which was consistent with the initial biopsy The oncology team, inspired by recent advances and promising results of clinical trials reported by Wang et al. in 2023, incorporated a combination of Taxol, carboplatin, atezolizumab, and Avastin into her treatment protocol [7]. This multifaceted approach was designed to attack cancer utilizing differing mechanisms of action in a synergistic attempt to curtail its spread and improve clinical outcomes. By June 2023, these efforts led to a substantial 55% reduction in the measurable extent of her disease, marking a significant, albeit temporary, victory in her ongoing battle with cancer (Figure 1).

Despite these aggressive therapeutic interventions, within three months, the relentless nature of her cancer led to persistent and worsening bony metastatic disease of her skull, spine, right rib, and femur, eventually necessitating a shift in focus toward palliative care. This phase was aimed at optimizing her quality of life and managing symptoms effectively, rather than continuing to pursue systemic therapeutic strategies. Her care during this period was characterized by a compassionate and supportive approach, tailored to her needs and comfort. Over time, however, her condition gradually worsened, and she peacefully passed away, surrounded by her loved ones. Her journey through lung cancer treatment underscores the complex, multifaceted challenges faced in managing advanced cancer, particularly in the context of personalized oncological care in a precision medicine landscape.

## Discussion

This case illustrates the potential limitations of osimertinib in treating bone metastasis in patients with EGFR-mutated lung adenocarcinoma. While the drug shows high efficacy in treating primary lung tumors and non-bone metastatic sites, its impact on bone metastasis is less effective, echoing findings from various studies [4,8]. Research, including a retrospective examination of clinical data, shows that bone metastasis can independently predict reduced progression-free survival (PFS) in patients treated with EGFR-TKIs, including osimertinib [9].

This suggests that alternative treatment strategies, such as combination therapies or new targeted treatments, might be necessary for these patients [10]. Patients with osimertinib resistance might benefit from combination therapies like afatinib and osimertinib, which have been shown to provide temporary disease control [3]. There is evidence in the literature supporting the idea that afatinib has more long-lasting PFS benefits than osimertinib [7]. Moreover, cerebrospinal fluid analysis can offer unique insights into resistance mechanisms, especially in cases with leptomeningeal metastases, suggesting that targeted therapies based on detailed genetic profiling could be beneficial post-resistance.

The primary resistance observed in some patients previously treated with other EGFR-TKIs indicates the complexity of managing resistance, which can include various factors such as mutation dynamics and previous treatments. Research indicates that the type of mutation significantly influences the timing of resistance development to osimertinib and whether osimertinib is employed as first-line or second-line therapy causes the incidence of resistance development to vary among patient populations [11,12]. Given the abundance of uncertainties surrounding osimertinib resistance, this case and other similar ones underscore the urgent requirement for comprehensive approaches to recognize and address osimertinib resistance, particularly in cases of bone metastases [8]. To elaborate further, it is essential to emphasize the multifaceted nature of treatment resistance in EGFR-mutated lung adenocarcinoma, particularly in bone metastases. Bone metastasis presents unique challenges due to the distinct microenvironment of the bone, which may influence the efficacy of medications like osimertinib. The bone matrix and its interactions with metastatic cancer cells can create a protective niche that allows cancer cells to evade the effects of EGFR-TKIs [9].

Moreover, studies have indicated that the bone microenvironment may promote different signaling pathways and genetic mutations that contribute to drug resistance [13]. This understanding highlights the importance of developing targeted therapies that not only address the primary tumor but also the specific conditions within the bone metastatic sites. Combining osimertinib with other agents, such as afatinib, has shown promise in overcoming resistance, albeit temporarily [14]. This suggests a potential strategy of sequential or combination therapy to extend the period of disease control. However, the long-term benefits and optimal combination strategies need further investigation through clinical trials.

Additionally, the role of detailed genetic and molecular profiling in guiding treatment decisions cannot be overstated. By analyzing cerebrospinal fluid and other biomarkers, clinicians can gain insights into the specific resistance mechanisms at play, allowing for a more personalized approach to therapy [15]. This precision medicine approach could lead to the development of new drugs or the repurposing of existing drugs to target the unique aspects of bone metastases in lung cancer. Recently, Chen et.al (2024) found that while the presence of bone metastasis is a negative prognostic factor for EGFR-mutated lung cancer patients, the addition of antiangiogenic therapy and denosumab, with sequential osimertinib can improve survival outcomes [16].

## Conclusions

This case underscores the challenges in treating bone metastasis in EGFR exon 19-mutated lung adenocarcinoma with osimertinib. Despite favorable initial responses, the ineffectiveness of the drug on bone lesions necessitates further research into more effective treatment strategies. The findings from this case contribute to the literature suggesting that personalized, perhaps multimodal, treatment strategies are essential for managing this complex disease.

## References

[1] Li C, Lei S, Ding L (2023). Global burden and trends of lung cancer incidence and mortality. Chin Med J (Engl).

[2] Soria JC, Ohe Y, Vansteenkiste J (2018). Osimertinib in untreated EGFR-mutated advanced non-small-cell lung cancer. N Engl J Med.

[3] Gu H, Sun L, Dou Z (2020). Analysis of lung adenocarcinoma with bone metastasis: a case report. Transl Lung Cancer Res.

[4] Zheng LP, Chen LY, Liao XY, Xu ZH, Chen ZT, Sun JG (2018). Case report: primary resistance to osimertinib in erlotinib-pretreated lung adenocarcinoma with EGFR T790 M mutation. BMC Cancer.

[5] Brouns AJ, van Veelen A, Veerman GD (2023). Incidence of bone metastases and skeletal-related events in patients with EGFR-mutated NSCLC treated with osimertinib. JTO Clin Res Rep.

[6] Ramalingam SS, Vansteenkiste J, Planchard D (2020). Overall survival with osimertinib in untreated, EGFR-mutated advanced NSCLC. N Engl J Med.

[7] Wang C, Zhao K, Hu S, Dong W, Gong Y, Xie C (2023). Clinical outcomes of afatinib versus osimertinib in patients with non-small cell lung cancer with uncommon EGFR mutations: a pooled analysis. Oncologist.

[8] Kanaoka K, Sumikawa H, Oyamada S (2023). Osteoblastic bone reaction in non-small cell lung cancer harboring epidermal growth factor receptor mutation treated with osimertinib. BMC Cancer.

[9] Wang M, Xia F, Wei Y, Wei X (2020). Molecular mechanisms and clinical management of cancer bone metastasis. Bone Res.

[10] Kim HS, Lim KY, Lee SH, Kim HY, Lee Y, Han JY (2023). Dynamics of disease progression during treatment with Osimertinib in patients with EGFR T790M-positive non-small cell lung cancer. Cancer Med.

[11] Gen S, Tanaka I, Morise M (2022). Clinical efficacy of osimertinib in EGFR-mutant non-small cell lung cancer with distant metastasis. BMC Cancer.

[12] Mu Y, Hao X, Xing P (2020). Acquired resistance to osimertinib in patients with non-small-cell lung cancer: mechanisms and clinical outcomes. J Cancer Res Clin Oncol.

[13] Yang C, Tian Y, Zhao F, Chen Z, Su P, Li Y, Qian A (2020). Bone microenvironment and osteosarcoma metastasis. Int J Mol Sci.

[14] Miura S, Koh Y, Azuma K (2023). Afatinib plus osimertinib in the treatment of osimertinib-resistant non-small cell lung carcinoma: a phase I clinical trial. BMC Cancer.

[15] Das S, Dey MK, Devireddy R, Gartia MR (2023). Biomarkers in cancer detection, diagnosis, and prognosis. Sensors (Basel).

[16] Chen WC, Cheng WC, Chen CL (2024). Assessing EGFR-mutated NSCLC with bone metastasis: clinical features and optimal treatment strategy. Cancer Med.

