# Correction: The Ineffectiveness of Osimertinib in Epidermal Growth Factor Receptor (EGFR)-Mutated Stage IV Lung Adenocarcinoma With Bone Metastasis: A Case Report

**DOI:** 10.7759/cureus.c188

**Published:** 2024-08-09

**Authors:** Caitlyn H Livingston, Benjamin Harper, Mathew George

**Affiliations:** 1 Neuroscience, Texas A&M University, College Station, USA; 2 Medical Oncology, Calvary Mater, Newcastle, AUS; 3 Medical Oncology, Calvary Mater Newcastle, Waratah, AUS

This article has been corrected to include the third author, Dr. Mathew George, who was erroneously omitted from the author list by the submitting author.

